# Assessment of medication adherence, medication safety awareness and medication practice among patients with lung cancer: A multicentre cross‐sectional study

**DOI:** 10.1111/hex.13426

**Published:** 2022-01-05

**Authors:** Ningsheng Wang, Biqi Ren, Haisheng You, Yue Chen, Shuzhi Lin, Shuang Lei, Bianling Feng

**Affiliations:** ^1^ The Department of Pharmacy Administration and Clinical Pharmacy, School of Pharmacy Xi'an Jiaotong University Xi'an Shaanxi China; ^2^ The Center for Drug Safety and Policy Research Xi'an Jiaotong University Xi'an Shaanxi China; ^3^ The First Affiliated Hospital of Xi'an Jiaotong University Xi'an Shaanxi China

**Keywords:** lung cancer, medication safety, patient awareness, patient participation

## Abstract

**Objectives:**

We aimed to explore the current status of medication adherence, safety awareness and practice among patients with lung cancer.

**Methods:**

We conducted a questionnaire‐guided cross‐sectional study in Xi'an, Yulin, Hanzhong and Weinan in Shaanxi Province, China, from April to June 2021 for a period of 3 months. The study questionnaire was developed according to previous related studies reported in the literature, and includes basic demographic information and patients' medication safety questions. The data were double‐entered using EpiData 3.1 software; descriptive statistics, *t*‐test, analysis of variance, the Kruskal–Wallis test and the Mann–Whitney *U*‐test were performed to analyse the data.

**Results:**

A total of 567 participants were included, and 409 valid questionnaires were finally completed, with an effective response rate of 72.13%. More than 80% of patients showed good medication adherence; the average adherence score was 22 ± 2.68 of 25. The average score for medication safety awareness was 16.40 ± 4.41, which was significantly lower than that of medication adherence (*p* < .001). Only 22.74% of patients always checked their medicines before a nurse administered them; 17.60% of patients never checked their medicines. Few patients actively consulted an health care professional to understand safety information before taking a medication. A significant difference existed in safety awareness scores among age groups (*p* = .039) and geographic regions (*p* < .001). Patients with three or more comorbidities had the lowest awareness scores (*p* = .027).

**Conclusion:**

We found that patients with lung cancer showed better medication adherence, but their awareness about medication safety was poor. Older patients, those with comorbidities and patients in regions with poor medical resources may have worse awareness about safety. Current medication education for patients should not only aim to improve adherence but should also encourage patients to take greater responsibility for their own safety and to actively participate in their medication safety. Greater systematic and individualized medication safety information is needed for older patients, those with more comorbidities and patients in areas with poor medical resources.

**Patient Contribution:**

We conducted a questionnaire‐guided cross‐sectional study on hospitalized lung cancer patients in Shaanxi Province to explore the patients' practices related to safety medication, including medication adherence and medication safety awareness.

## 1 INTRODUCTION

Cancer is the second most common cause of death worldwide, and the number of cancer cases and deaths has been increasing each year.^1^ The latest global cancer data^2^ released by the International Agency for Research on Cancer (IARC) of the World Health Organization (WHO) show that in 2020, there were 9.96 million cancer deaths worldwide, including 1.8 million lung cancer deaths, far surpassing other cancer types and ranking first in terms of the number of cancer deaths.

The treatment of cancer mainly involves surgical treatment, radiotherapy and drug treatment.^3^ Various treatment methods complement each other to yield better treatment results. With the development of medical technology and new anticancer drugs, medication has become the main method of comprehensive anticancer treatment.^3^, ^4^ However, drug safety is important, given that most anticancer drugs have a narrow therapeutic index and individual differences in toxicity,^5^ and the chemotherapy regimen is complex and involves many combined drugs. At the same time, the physiological function of important organs, immune function and pharmacokinetic characteristics are easily affected by disease progression in patients with cancer,^6^ and drugs are mostly administered to older patients with cancer and multiple comorbidities,^5^, ^7^ which leads to patients with cancer having a considerably higher risk of adverse drug reactions (ADRs) and serious adverse events, and there is also the potential for serious medical errors.^7^, ^8^ A retrospective analysis of mortality related to medication errors showed that the use of anticancer drugs is the second most common cause of death.^9^ Most antitumor drugs, such as platinum‐based drugs and antimetabolites, are cytotoxic drugs. These types of drugs have poor targeting; while killing tumour cells, they inevitably exert a toxic effect on normal tissues and organs, the drug therapeutic window is narrow, drug doses must be calculated according to body weight and each medication must be administered at precisely maintained time intervals, so any carelessness in the administration process can lead to medication errors.^7^, ^10^ Medication errors may cause toxic side effects, hospitalization and even death. Therefore, medication safety in anticancer treatment is very important for patients with cancer.^5^, ^7^

Cancer treatment is complex and involves multidisciplinary teams. There are many safety risk factors in treatment for cancer.^11^ These risks occur at multiple key points, from prescription to deployment to drug administration. Therefore, it is necessary to coordinate the efforts of health care professionals (HCPs) to minimize risk.^12^, ^13^ At present, many studies are aimed at certain types of HCPs, exploring potential factors that affect the safety of patients' medication in their work processes so as to propose specific intervention measures to ensure the safety of patients' medication. For example, from the perspective of nurses,^14^, ^15^, ^16^ it has been shown that risk factors related to patient medication safety include nurse medication errors, poor communication, unclear doctor's orders, heavy workload and personnel rotation. From the perspective of pharmacy‐related professionals,^17^, ^18^, ^19^ it has been shown that factors such as a lack of clinical collaboration and lack of pharmacy services affect patients' medication safety. These studies can serve as a reference and guidance for medication safety in patients with cancer from the perspective of HCPs. As the individual with the most direct contact with the drug, the patient represents a key factor affecting the safe use of his or her medication in cancer treatment.^20^ The value of allowing patients and their families to participate in medication safety has been recognized in health care worldwide.^21^ One of the primary initiatives derived from the patient safety movement is to approach patient participation as a patient safety strategy.^22^, ^23^

Studies have shown^24^, ^25^, ^26^, ^27^ that HCPs believe that patients' participation in their medication safety mainly involves medication adherence. Therefore, many studies have adopted structured intervention guidelines to improve medication adherence among patients with cancer to ultimately improve patient outcomes.^20^, ^28^ In addition to medication adherence, the patient's medication practices and medication safety awareness will affect the safe use of their medication. On the premise that patients have a better awareness of medication safety, patients are more likely to have better adherence and medication practice. However, there are currently few studies on patients' medication safety awareness and practices. Therefore, the purpose of this study was to explore the current status of medication adherence, medication safety awareness and medication practice among patients with lung cancer, to provide a research basis for subsequent targeted interventions to help patients improve their safe medication use.

## 2 METHODS

### 2.1 Study area and study population

According to the 2020 Statistical Yearbook of Shaanxi Province,^29^ the population of Shaanxi Province in 2019 was 38.75 million, ranking 16th out of 31 provinces in China and fourth out of 12 provinces in the west of the country. The per capita GDP of Shaanxi Province in 2019 was ranked 12th nationally and third among the 12 western provinces. In terms of geographic location, population size and economic conditions, Shaanxi Province is representative of the 12 provinces in western China.

We evaluated the geographic environment and economic development of various regions in Shaanxi Province. The province is divided into three regions (Northern, Central and Southern Shaanxi) and one provincial capital city (Xi'an). Taking the median per capita GDP of 11 regions in Shaanxi Province as a benchmark, in addition to the provincial capital city (Xi'an), we selected Yulin (120,900 RMB, high per capita GDP), Hanzhong (45,000 RMB, medium per capita GDP) and Weinan (34,500 RMB, low per capita GDP) as representative cities. We included general hospitals with an oncology department or cancer specialist hospitals in each city as the research sites. In these hospitals, simple random sampling methods were used to select patients with lung cancer as the research participants. The specific method of sampling and questionnaire distribution were as follows: a catalogue of currently hospitalized lung cancer patients was obtained from the Hospital Information System, each patient was numbered and these patient numbers were input into SPSS. Simple random sampling was conducted using the function of ‘Data → Select Cases→Random sample of Cases’ of SPSS software, 10–15 patients were randomly selected each time and we recorded the ward and bed number of these randomly sampled lung cancer patients. Then, we went to the ward and invited the patient to participate in the study. A face‐to‐face questionnaire survey was conducted in our investigation. We recommended that competent patients fill out the questionnaire by themselves; older patients gave their responses verbally to study staff, who recorded these. The questionnaire was distributed to all participants, accompanied by an explanatory letter informing them of the purpose of the survey. The content of the questionnaire did not involve the personal information of the respondent, so anonymity was guaranteed.

We conducted this questionnaire‐guided cross‐sectional study in Xi'an, Yulin, Hanzhong and Weinan in Shaanxi Province from April to June 2021 for a period of 3 months. We used the Raosoft sample size calculator^30^ to calculate the sample size. According to the incidence of lung cancer in China^31^ during 2015 (57.26/100,000) and the total population of Shaanxi Province, there are approximately 22,000 patients with lung cancer in Shaanxi Province. We assumed a 95% confidence level and a 50% acceptable margin of error; the response distribution was 50%. The calculated sample size was 378 participants, to which we added 50% to cover possible invalid questionnaires, such as those with logical inconsistencies. We finally determined that 567 participants were needed. The number of participants in each selected area was estimated using the population ratio (Xi'an: 10.20 million; Yulin: 3.42 million; Hanzhong: 3.44 million; Weinan: 5.28 million).

### 2.2 Questionnaire design

We developed the study questionnaire according to previous related studies reported in the literature.^32^, ^33^ The questionnaire consists of two parts. The first part contained basic demographic information, including sex, age, number of comorbidities, length of treatment, family history of cancer and history of smoking. The second part addressed patients' medication safety, which mainly included (1) medication adherence, (2) awareness of medication safety and (3) other medication practices. The options for the medication adherence and medication safety awareness parts of the questionnaire were scored using a 5‐point scale. The five questions on medication adherence were assigned points as follows: Questions 1–3: ‘always’ = 1 point, ‘usually’ = 2 points, ‘generally’ = 3 points, ‘rarely’ = 4 points and ‘never’ = 5 points; Questions 4 and 5: ‘always’ = 5 points, ‘usually’ = 4 points, ‘generally’ = 3 points, ‘rarely’ = 2 points and ‘never’ = 1 point. The five questions on awareness about medication safety were assigned points as follows: 1–5 questions: ‘always’ = 5 points, ‘usually’ = 4 points, ‘generally’ = 3 points, ‘rarely’ = 2 points and ‘never’ = 1 point. The score range for both these parts was 5–25 points; the higher the score, the better the patient's adherence or awareness about medication safety.

To ensure participant recruitment procedures and validity and reliability of the measuring instrument, we conducted expert interviews and a pilot study before initiating the main study. The developed questionnaire was verified for readability and acceptability among five HCPs who were working in a grade‐A tertiary hospital. To ensure the professionalism of the experts, we established the following inclusion criteria: working in a grade‐A tertiary hospital, clinically engaged in the treatment of patients with lung cancer, more than 15 years of work experience and having a professional vice‐senior title or above. After the experts had modified the questionnaire, we conducted a pilot study including 20 people. The contents of the questionnaire were modified according to the pilot study. The findings of the pilot study were not included in the final data analysis.

### 2.3 Data analysis

After excluding invalid questionnaires, all valid questionnaires were numbered and the data were double‐entered using EpiData 3.1 software. All statistical analyses were performed using Microsoft Office Excel 2016 (Microsoft Corporation) and IBM SPSS 24.0 (IBM Corp.). We performed descriptive statistics on the demographic characteristics of the patients and their responses for each question. We used the *t*‐test, analysis of variance, the Kruskal–Wallis test and the Mann–Whitney *U*‐test to test the differences in patient medication adherence and medication safety awareness with different demographic characteristics. Statistical significance was defined as *p* < .05.

## 3 RESULTS

### 3.1 Demographic characteristics

A total of 567 questionnaires were distributed. After excluding invalid questionnaires, 409 valid questionnaires were finally included in this study, with an effective response rate of 72.13%. Cronbach's *α* value was calculated to measure the internal consistency, which was found to be .72, signifying good internal consistency.

Two thirds (66.99%) of patients with lung cancer were men. Most patients (88.26%) were older than 50 years of age, with most patients in the age group of 61–70 years (36.67%). The proportion of patients older than 70 years of age reached 21.27%. In total, 31.54% of patients had no comorbidities apart from lung cancer. Among the 67.97% of patients with comorbidities, 67.99% of patients had one or two comorbidities and 32.01% had three or more comorbidities. A total of 5.62% of patients did not know how many medications they were taking, 26.16% of patients used more than five drugs in combination and 21.27% of patients used three drugs in combination. The proportion of patients with anticancer treatment duration of less than 1 year was 75.06%; more than half of the patients (57.95%) reported having ADRs during anticancer treatment, 11.98% had a family history of cancer, 41.08% had no history of smoking and 41.81% had a history of smoking for 20 years (Table 1).

**Table 1:** Demographic characteristics among 409 patients with lung cancer in Shaanxi, China (2021)

Characteristic	Number	Percentage	Characteristic	Number	Percentage
Sex			Duration of treatment		
Male	274	66.99%	<1 month	77	18.83%
Female	133	32.52%	1–3 months	90	22.00%
No data	2	0.49%	3–6 months	56	13.69%
Age, years			6 months to 1 year	84	20.54%
18–40	14	3.42%	1–3 years	77	18.83%
41–50	34	8.31%	3–5 years	13	3.18%
51–60	124	30.32%	>5 years	12	2.93%
61–70	150	36.67%	Family history of cancer		
>71	87	21.27%	Yes	49	11.98%
Comorbidities			No	322	78.73%
1 or more comorbidities	278	67.97%	Unsure	38	9.29%
No comorbidities	129	31.54%	Smoking history		
No data	2	0.49%	No smoking history	168	41.08%
Number of comorbidities			<1 year	1	0.24%
1	104	37.41%	1–5 years	12	2.93%
2	85	30.58%	5–10 years	15	3.67%
3	58	20.86%	10–20 years	42	10.27%
4	19	6.83%	>20 years	171	41.81%
5	5	1.80%	Quit smoking^a^		
>5	7	2.52%	No	23	9.54%
Number of combined drugs			Yes	212	87.97%
Unsure	23	5.62%	No data	6	2.49%
1	19	4.65%	Quit smoking attitude^a^		
2	47	11.49%	Strongly agree	147	61.00%
3	87	21.27%	Agree	52	21.58%
4	74	18.09%	Not necessarily	26	10.79%
5	52	12.71%	Disagree	4	1.66%
>5	107	26.16%	Strongly disagree	6	2.49%
ADRs during cancer treatment			No data	6	2.49%
Yes	237	57.95%			
No	170	41.56%			
No data	2	0.49%			

Abbreviation: ADR, adverse drug reaction.

^a^ These two questions were only completed by 241 patients with a history of smoking.

### 3.2 Patient medication adherence

Table 2 shows that only 37.65% of the included patients stated that they never forget to take their medication during treatment; 55.99% never increased or decreased the dosage of medication by themselves; 63.33% never adjusted the infusion rate by themselves; and 65.04% and 64.03% of patients took their medications as planned during hospitalization and treatment at home, respectively.

**Table 2:** Medication adherence and safety awareness among patients with lung cancer in Shaanxi, China (2021)

	Number (%)				
	Always	Usually	Generally	Rarely	Never
*Medication adherence*					
Q1 Do you forget to take your medicine during treatment?	10 (2.44)	15 (3.67)	37 (9.05)	193 (47.19%)	154 (37.65%)
Q2 Do you increase or decrease the dose or stop the medication yourself?	6 (1.47)	15 (3.67)	28 (6.85)	131 (32.03)	229 (55.99)
Q3 Do you adjust the infusion rate yourself?	2 (0.49)	16 (3.91)	31 (7.58)	101 (24.69)	259 (63.33)
Q4 During hospitalization, do you adhere to the treatment plan and take your medications on time and at the prescribed doses?	266 (65.04)	105 (25.67)	21 (5.13)	12 (2.93)	5 (1.22)
Q5 Do you take your medicine on time and at the proper dose during treatment at home after discharge from the hospital?	263 (64.30)	109 (26.65)	27 (6.60)	6 (1.47)	4 (0.98)
*Medication safety awareness*					
Q1 Do you check your medicine before the nurse administers it?	93 (22.74)	76 (18.58)	63 (15.40)	105 (25.67)	72 (17.60)
Q2 After taking your medicine, do you actively watch for any reactions?	90 (22.00)	116 (28.36)	88 (21.52)	81 (19.80)	34 (8.31)
Q3 Do you take the initiative to ask an HCP for medication safety information, such as about ADRs?	59 (14.43)	76 (18.58)	110 (26.89)	103 (25.18)	61 (14.91)
Q4 Do you take the initiative to check the medication insert for precautions, ADRs, storage conditions and other safety information before taking a medication?	72 (17.60)	85 (20.78)	95 (23.23)	103 (25.18)	54 (13.20)
Q5 Do HCPs inform you of ADRs, medication precautions and provide other safety information before administering a medication?	153 (37.41)	156 (38.14)	70 (17.11)	23 (5.62)	7 (1.71)

Abbreviations: ADRs, adverse drug reactions; HCP, health care professional.

The first three questions in the medication adherence section of the survey were negative response options; responses of ‘rarely’ and ‘never’ were considered to indicate good medication adherence. Questions 4 and 5 were positive response options; responses of ‘always’ and ‘usually’ indicated good adherence. The proportion of patients indicating good medication adherence was 84.84%, 88.02%, 88.02%, 90.71% and 90.95%, respectively (Table 2).

The average score for patient medication adherence was 22 ± 2.68 (full score 25), and the median score was 23. There were significant differences in medication adherence scores between patients in different geographic regions (*p* < .001). Patients in Xi'an had the highest scores and those in Yulin had the lowest scores. Pairwise comparisons showed that the differences in scores between patients in Xi'an and Yulin were statistically significant. The medication adherence scores of patients without comorbidities were higher than those of patients with comorbidities (*p* = .001), and the adherence scores of patients with one comorbidity were higher than those of patients with multiple comorbidities (*p* = .028). The adherence scores of patients in the group with three or more comorbidities were the lowest and were significantly different from the remaining two groups. The adherence scores of patients who did not have ADRs during the treatment period were higher than the scores among those who experienced ADRs (*p* = .024), and the difference was statistically significant (Table 3).

**Table 3:** Scores for medication adherence and medication safety awareness among patients with lung cancer in Shaanxi, China (2021)

		Medication adherence			Medication safety awareness		
Characteristic		Mean score	95% CI	*p*	Mean score	95% CI	*p*
Sex							
Male		21.92	21.59–22.24	.263	16.35	15.84–16.86	.646
Female		22.23	21.81–22.67		16.53	15.77–17.36	
Age (years)							
18–40		23.29	21.66–24.91	.404	19.79	18.21–21.36	.039*
41–50		22.15	21.25–23.06		16.12	14.76–17.48	
51–60		22.05	21.60–22.50		16.02	15.18–16.87	
61–70		21.93	21.46–22.41		16.46	15.77–17.15	
>70		21.78	21.27–22.29		16.39	15.46–17.32	
Region							
Xi'an		22.47	22.10–22.85	<.001*	17.47	16.85–18.10	<.001*
Weinan		21.49	21.01–21.98		16.45	15.70–17.20	
Hanzhong		22.58	21.89–23.27		15.33	14.24–16.43	
Yulin		20.70	20.00–21.41		14.03	12.90–15.16	
Comorbidities							
Yes		21.70	21.37–22.03	.001*	16.23	15.71–16.76	.204
No		22.62	22.22–23.02		16.83	16.08–17.58	
Number of comorbidities							
1		22.17	21.64–22.70	.028*	16.41	15.54–17.29	.027*
2		21.75	21.22–22.28		17.04	16.12–17.95	
>3		21.10	20.45–21.75		15.26	14.32–16.20	
Number of combined drugs							
1–2		22.33	21.74–22.93	.405	17.17	16.19–18.15	.289
3–4		21.83	21.39–22.26		16.15	15.44–16.86	
>5		22.07	21.65–22.49		16.49	15.79–17.19	
Duration of treatment							
<1 month		22.16	21.61–22.70	.496	15.62	14.58–16.66	.465
1–3 months		22.48	22.01–22.95		16.89	15.94–17.84	
3–6 months		21.57	20.68–22.45		16.56	15.42–17.69	
6 months to 1 year		21.86	21.22–22.49		16.48	15.48–17.47	
>1 year		21.81	21.29–22.33		16.40	15.60–17.21	
ADRs during cancer treatment							
Yes		21.73	21.39–22.07	.024*	16.41	15.67–17.15	.871
No		22.34	21.93–22.75		16.38	15.95–16.81	

Abbreviations: ADRs, adverse drug reactions; CI, confidence interval.

* Significant difference with a *p* value < .05.

### 3.3 Patients' awareness of medication safety

Table 2 shows that only 22.74% of patients reported always checking their medicines before the nurse administered them, and 17.60% of patients reported never checking their medicines; 22% of patients always observed their reactions after taking a medication during treatment (e.g., whether they had swelling, tenderness, tingling, burning sensations or other subjective abnormal sensations after drug infusion); 8.31% of patients never took note of any reactions after taking a medication. Few patients actively consulted an HCP so as to understand safety information before taking a medication; only 14.43% of patients responded that they ‘always’ actively consulted an HCP. A small proportion of patients actively checked the information regarding medication precautions, ADR, drug shelf‐life, production date, storage conditions and other medication safety information in the medication package insert. Patients who responded ‘always’ or ‘usually’ to the five questions on medication safety awareness were considered to have better awareness and understanding, with the proportions of patients with better awareness and cognition for each question in this part of the survey being 41.32%, 50.37%, 33.01%, 38.39% and 75.55%.

The average score for medication safety awareness was 16.40 ± 4.41 (full score 25), and the median score was 16; the medication adherence score (22 ± 2.68) was higher than that for patient medication safety awareness, and the difference was statistically significant (*p* < .001). There was a significant difference in safety awareness scores among different age groups (*p* = .039). In pairwise comparison, we found that patients in the age group of 18–40 years had the highest scores, which were significantly different from the remaining groups. There were statistically significant differences in the medication safety awareness scores of patients in different geographic regions (*p* < .001), with the highest scores in Xi'an and the lowest scores in Yulin. Patients with three or more comorbidities had the lowest scores (*p* = .027); in pairwise comparison, we found that scores in this group were significantly different from those of other groups who had fewer comorbidities (Table 3).

### 3.4 Patients' other medication safety practices

Table 4 shows that two thirds of patients (66.99%) knew the correct way of taking each medicine and could take their medicines by themselves; however, 31.54% of patients needed a reminder or the help of others to take their medicine. When a dose of medication was missed, one third of patients (30.81%) reported that they took the next planned dose on time. Most patients reported taking the initiative to inform HCPs about a history of drug allergies and ADRs. With medication problems such as worsening symptoms or other adverse reactions during treatment, most patients (53.79%) reported consulting with the physician in charge; only 0.73% of patients consulted a pharmacist, and 6.36% reported ignoring ADRs. When asked about their preferred channels for obtaining medication safety information, most patients (75.06%) were more likely to obtain medication information via public health educational information provided by HPCs; 16.14% of patients tended to use WeChat push notifications, QQ groups, SMS reminders, TV, radio and other multimedia channels to obtain information on medication safety (Table 4).

**Table 4:** Medication practice among patients with lung cancer in Shaanxi, China (2021)

	Number (%)
*Q1 Do you know how and when to take your oral medications?*	
I know the way and time to take each medicine, and I can take the medicine by myself	274 (66.99)
I know this somewhat, but still need a reminder and help from a caregiver or HCP to take the medicine	109 (26.65)
I don't know this at all, and I need reminders or help from caregivers or HCPs to take my medication	20 (4.89)
No data	6 (1.47)
*Q2 Do you know what to do if you miss a dose of medicine?*	
Add the missed dose to the next dose	10 (2.44)
When I notice that I missed a dose, I take it immediately	68 (16.63)
I never forget a dose of medication	105 (25.67)
Take the next planned dose on time	126 (30.81)
Ask for help from an HCP	75 (18.34)
Other	25 (6.11)
*Q3 If you have a history of drug allergies or ADR, do you actively tell an HCP?*	
I take the initiative to inform an HCP of the situation	303 (74.08)
I give this information after an HCP asks me about it	106 (25.92)
*Q4 During treatment, if symptoms worsen or other adverse reactions occur after medication, you*	
Consult a nurse	156 (38.14)
Consult the physician	220 (53.79)
Consult a pharmacist	3 (0.73)
Wait for the situation to improve, ignore the ADR	26 (6.36)
Stop or reduce the drug dose myself	4 (0.98)
*Q5 Which channel do you prefer to obtain medication safety information*	
Education on medication safety from HCPs	307 (75.06)
Multimedia channels such as WeChat push, QQ groups, SMS reminders, TV and radio	66 (16.14)
Medical information pamphlets, books, magazines and other printed materials	30 (7.33)
Other	6 (1.47)

Abbreviations: ADR, adverse drug reaction; HCP, health care professional.

## 4 DISCUSSION

In this study, Xi'an, Yulin, Hanzhong and Weinan in Shaanxi Province were taken as representative cities to investigate the current status and related factors of medication adherence, safety awareness and practice among patients with lung cancer. Among the 409 valid questionnaires, male patients accounted for two thirds of respondents. The latest global cancer data and China's epidemiological data^1^, ^2^ both show that both the number of incident cases and incidence rates of lung cancer are higher among male than female patients. Most of our respondents were elderly patients, among whom 21.27% were older than 70 years of age. Patients had a variety of comorbidities. Up to 26.16% of patients used more than five drugs in combination, and more than half (57.95%) experienced ADRs during treatment. One study^34^ showed that cancer disproportionately affects older people, with more than one third of cancers diagnosed in those over the age of 70 years. Most older adults have decreased liver and kidney function, weakened metabolic capacity, more comorbidities and a large number of coadministered drugs, so they are at higher risk of ADRs.^35^, ^36^ Ageing of the global population presents considerable challenges to the care planning of medication safety for patients with cancer. In this study, nearly 12% of patients had a family history of cancer. A Chinese study^37^ showed that among lung cancer patients, the proportion with a family history of lung cancer was 28%, and the proportion with a family history of cancer was 36.1%. A retrospective literature study^38^ found that the risk of lung cancer in patients with a family history of cancer was 2.47 times higher than that in patients without a family history of cancer. The incidence of cancer among family members of patients with cancer, especially lung cancer, is significantly higher than that among the general population.^39^ The occurrence of lung cancer is affected by genetic factors. Providing health education is important for the families of patients with lung cancer, and timely physical examination should be urged for the early detection and diagnosis of cancer. In our study, 40.81% of patients had a history of smoking for more than 20 years. Approximately 70% of lung cancer‐related deaths worldwide are associated with tobacco use, with smokers being 20 times more likely to die from lung cancer‐related conditions than their nonsmoking counterparts.^40^, ^41^ Smoking cessation has been identified as one of the most effective strategies to reduce the incidence of lung cancer.^42^ People with lung cancer should be urged to quit smoking as soon as possible.

The medication adherence of patients with lung cancer in this study was better than that of patients with other diseases reported in other studies. Previous studies have shown that the adherence rate among people with long‐term chronic diseases averages only 50%.^43^, ^44^ A study of patients with bipolar affective disorder showed that only 28.5% had good medication adherence.^45^ The scope of treatment with oncology drugs is narrow, and treatment interruption must be avoided.^46^ Therefore, nonadherence behaviours such as forgetting to take medicines among patients with cancer will lead to more serious consequences, affecting patient safety and impairing the treatment effect. A patient‐specific interview study^46^ showed that patients with cancer had good medication adherence; these patients stated that they need to be very responsible regarding their medication because the consequences of nonadherence to oncology drugs can be serious. This may be the reason for the relatively good adherence among patients with lung cancer in our study.

Patients in Xi'an had the highest scores for medication adherence and medication safety awareness, and patients in Yulin had the lowest scores. Xi'an is the capital city of Shaanxi Province, with richer medical resources and better economic development. According to the official data of Shaanxi Province,^29^ the number of health institutions, beds and medical technical personnel in Xi'an are much higher than those in Yulin. This study showed that patients in areas with more abundant medical resources will have better medication adherence and medication safety awareness. It may be that HCPs in areas with abundant medical resources are more professional; these HCPs may provide patients with better safety education and have better communication about treatment and medication. China has a vast territory, and its different regions have different levels of development. Medical resources in remote areas are scarce and unevenly distributed.^47^ Global data also show that developed countries have more abundant medical resources than underdeveloped regions in Africa.^48^ With the development of 5G communication technology, China and other countries are working hard to promote telemedicine for consultation, diagnosis, patient health education and other activities to address the uneven distribution of medical resources between regions and help to solve the shortage of medical resources.^49^ Patients with comorbidities have worse adherence than those with no comorbidities, and the adherence of patients with more comorbidities is worse than that of patients with fewer comorbidities. Several studies have shown that comorbidities are related to a lack of medication adherence.^45^, ^50^ This could be explained by the fact that patients with comorbidities are more likely to take multiple drugs; thus, it is unlikely that they will take all their drugs properly. Moreover, the risk of adverse drug events is increased in patients with comorbidities, which can also negatively affect the adherence rate.^50^ The present study also showed that patients who experienced ADRs during treatment had worse adherence than those who did not experience ADRs; patients tend to stop taking medication owing to the strong ADRs and side effects of chemotherapy.^46^ Strong ADRs after chemotherapy seriously affect patients' quality of life, which may be a major obstacle to adherence to drug therapy.^45^

In this study, patients' awareness about medication safety was poor, and their awareness score was significantly lower than their medication adherence score. A study among patients with chronic diseases showed that HPCs have better communication with patients regarding the purpose and methods of taking medication than about medication safety information.^51^ HCPs believe that patients' involvement in medication safety is mainly via improving their adherence^24^, ^25^, ^26^, ^27^; HCPs ignore the fact that patients can participate in monitoring their own medication, reporting ADRs and can actively learn safety information. Patients' participation in their own medication safety plan is currently a major theme on the international patient safety agenda.^24^ In addition to improving medication adherence for the purpose of patient education, patients should also be encouraged to actively participate in their own medication safety and improve their medication safety awareness, such as checking one's own medicines, reading the package inserts of medicines, actively monitoring ADRs after drug administration and actively reporting ADRs. Studies have also shown that younger patients have better awareness about medication safety. This suggests that public health educational information should be simple and effectively adapted to elderly patients, such as by use of larger font print, avoiding the use of complex medical terms and using more graphics rather than a large number of words, among other approaches.^51^

More than half of the patients reported that they consulted with a physician when they experienced worsening of symptoms after taking a medication, which is the same as in another study.^51^ Only 0.73% of patients consulted a pharmacist about medication‐related issues. This may be related to the slow development of the field of clinical pharmacy in China, the insufficient number of clinical pharmacists, the difficulty in meeting the needs of ward and outpatient pharmacy services and low awareness about clinical pharmacists among the public.^52^ Therefore, with the reform of China's medical and health system, clinical pharmacists can participate more in clinical work, provide patients with more comprehensive pharmaceutical services and reduce the communication pressure between doctors and patients.^52^ In this study, 6.36% of patients stated that they ignored ADRs. This may be because patients hold the notion that a patient must ‘endure’ the side effects of chemotherapy for it to be effective.^46^ Patients should be encouraged to seek help from HCPs in case of ADRs and side effects so as to determine whether adjustment of the dose or the dosing regimen is necessary. The vast majority of patients (75.6%) stated that they preferred to obtain information about medication safety via public health education from HCPs, and 21.17% of patients believed that HCPs need to strengthen patient safety education. At the same time, some patients (16.14%) stated that they prefer to obtain information via multimedia channels such as WeChat and SMS. Studies^25^, ^46^, ^53^, ^54^ have reported that patients prefer to obtain information from printed materials, such as hospital health manuals and posters, or to search for relevant information on the Internet. HCPs can provide educational information via the use of apps and patient portals, among other avenues.

### 4.1 Strengths and limitations

This study was a rare medication safety survey conducted from the perspectives of patients' medication adherence, medication safety awareness and medication practice, aiming to explore those factors that affect medication safety among patients. Previous studies on patient participation in their own medication safety have mostly been intervention studies on patient adherence.^24^, ^25^, ^26^, ^27^ This study supplements other studies that have mostly only focused on adherence. The limitation of this study lies in the representativeness of the sample in our research. To ensure a representative sample size, the corresponding statistical formula was used to calculate the sample size; simple random sampling was carried out according to the number of participants needed to avoid selection bias. We included inpatients with lung cancer in Shaanxi Province, a representative province in western China; the basic situation of patients with lung cancer in other provinces in eastern and central China must be further investigated using a larger and more representative sample. Additionally, this was a cross‐sectional study. The design of this study can reflect the state of patients at a certain stage, but it cannot prove causality. Also, information about patients' adherence and awareness about medication safety was provided by patients, which may cause the findings to be unreliable owing to patient recall in self‐reporting, and it is possible that people might have been less comfortable providing negative responses to a researcher directly, which may affect the results.

## 5 CONCLUSIONS

Our investigation of medication adherence, medication safety awareness and medication practices revealed the limitations and obstacles to self‐participation in medication safety among patients with lung cancer. This study showed that patients with lung cancer had better medication adherence, but their awareness about medication safety was poor, and patients were unaware of the importance of participation in their medication safety. At the same time, elderly patients, patients with comorbidities, those with a greater number of comorbidities and patients in areas with poor medical resources may have worse adherence and safety awareness. Therefore, current medication education for patients should aim not only to improve adherence but should also encourage patients to take greater responsibility for their own safety and to actively participate in their safe medication use. Various channels, such as safety education from HPCs and multimedia, can serve to improve patients' safety awareness and medication adherence and protect their medication safety. At the same time, there should be more systematic and individualized publicity and safety education for elderly patients, patients with more comorbidities and those in areas with poor medical resources. This study can provide a research basis for better formulation of targeted patient medication safety intervention measures.

## CONFLICT OF INTERESTS

The authors declare that there are no conflict of interests.

## ETHICS STATEMENT

Approval for this study was received from the medicine and biomedical ethics committee of Xi'an Jiaotong University (No. 2021‐1342). Informed consent was obtained from all individual participants included in the study.

## AUTHOR CONTRIBUTIONS

Bianling Feng, Ningsheng Wang and Haisheng You conceived and designed the study; Ningsheng Wang, Yue Chen, Biqi Ren and Haisheng You performed the data analyses and conducted the questionnaire survey; Ningsheng Wang and Haisheng You designed the questionnaire; Ningsheng Wang wrote the manuscript; Shuzhi Lin and Shuang Lei provided constructive suggestions for the revision of the manuscript. Bianling Feng helped perform the analysis with constructive discussions and revised it critically for important intellectual content; Bianling Feng gave the final approval of the version to be published, and all authors agree to be accountable for all aspects of the work.

## Data Availability

All data generated or analysed during this study are included in this published article.
